# Supplementary material for: Comprehensive Annotation and Functional Exploration of MicroRNAs in Lettuce
Source: Front Plant Sci. 2021 Dec 24;12:781836. doi: 10.3389/fpls.2021.781836 (PMC8739914; doi:10.3389/fpls.2021.781836)
Supplement: Supplementary file 2 [file Data_Sheet_8.ZIP › GSM2230749.plot/Lsa-miR157b_Lsat_1_v5_gn_7_11040.1_1088_TPlot.pdf]

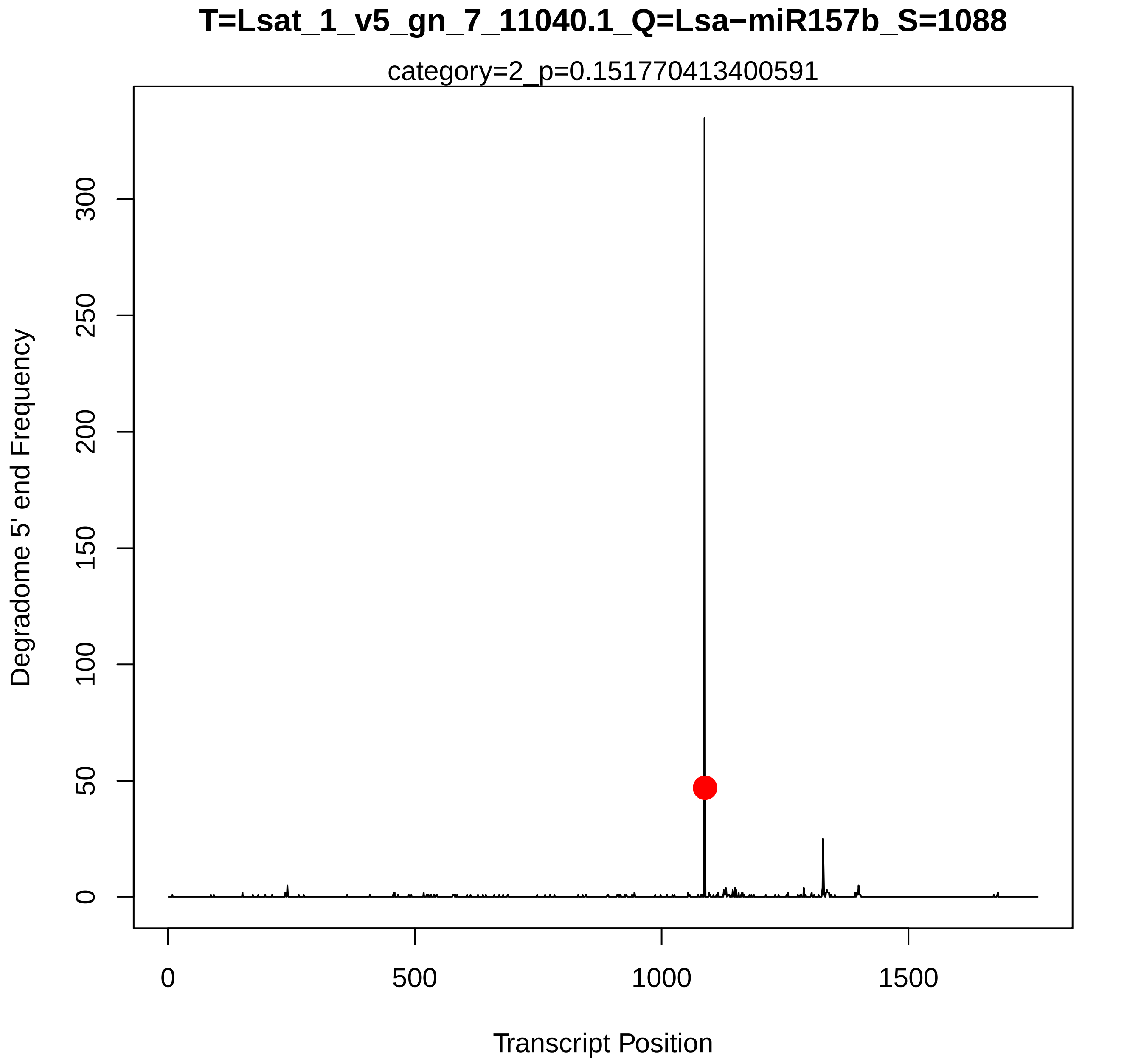
T=Lsat_1_v5_gn_7_11040.1_Q=Lsa−miR157b_S=1088
category=2_p=0.151770413400591
Degradome 5' end Frequency
Transcript Position
0
50
100
150
200
250
300
0
500
1000
1500
